# Personalized prediction of one-year mental health deterioration using adaptive learning algorithms: a multicenter breast cancer prospective study

**DOI:** 10.1038/s41598-023-33281-1

**Published:** 2023-04-29

**Authors:** Konstantina Kourou, Georgios Manikis, Eugenia Mylona, Paula Poikonen-Saksela, Ketti Mazzocco, Ruth Pat-Horenczyk, Berta Sousa, Albino J. Oliveira-Maia, Johanna Mattson, Ilan Roziner, Greta Pettini, Haridimos Kondylakis, Kostas Marias, Mikko Nuutinen, Evangelos Karademas, Panagiotis Simos, Dimitrios I. Fotiadis

**Affiliations:** 1 Unit of Medical Technology and Intelligent Information Systems, Dept. of Materials Science and Engineering, University of Ioannina, Ioannina, Greece; 2 Foundation for Research and Technology-Hellas, Biomedical Research Institute, Ioannina, Greece; 3 Foundation for Research and Technology-Hellas, Institute of Computer Science, Heraklion, Greece; 4 Helsinki University Hospital Comprehensive Cancer Center and Helsinki University, Helsinki, Finland; 5 Applied Research Division for Cognitive and Psychological Science, European Institute of Oncology IRCCS, Milan, Italy; 6 Dept. of Oncology and Hemato-Oncology, University of Milan, Milan, Italy; 7 School of Social Work and Social Welfare, The Hebrew University of Jerusalem, Jerusalem, Israel; 8 Breast Unit, Champalimaud Clinical Centre, Champalimaud Foundation, Lisbon, Portugal; 9 Champalimaud Research and Clinical Centre, Champalimaud Foundation, Lisbon, Portugal; 10 NOVA Medical School, NMS, Universidade Nova de Lisboa, Lisbon, Portugal; 11 Dept. of Communication Disorders, Sackler Faculty of Medicine, Tel Aviv University, Tel Aviv, Israel; 12 Nordic Healthcare Group, Helsinki, Finland; 13 Dept. of Psychology, University of Crete, Rethymno, Greece; 14 School of Medicine, University of Crete, Heraklion, Greece

**Keywords:** Biomedical engineering, Quality of life, Breast cancer

## Abstract

Identifying individual patient characteristics that contribute to long-term mental health deterioration following diagnosis of breast cancer (BC) is critical in clinical practice. The present study employed a supervised machine learning pipeline to address this issue in a subset of data from a prospective, multinational cohort of women diagnosed with stage I–III BC with a curative treatment intention. Patients were classified as displaying stable HADS scores (Stable Group; n = 328) or reporting a significant increase in symptomatology between BC diagnosis and 12 months later (Deteriorated Group; n = 50). Sociodemographic, life-style, psychosocial, and medical variables collected on the first visit to their oncologist and three months later served as potential predictors of patient risk stratification. The flexible and comprehensive machine learning (ML) pipeline used entailed feature selection, model training, validation and testing. Model-agnostic analyses aided interpretation of model results at the variable- and patient-level. The two groups were discriminated with a high degree of accuracy (Area Under the Curve = 0.864) and a fair balance of sensitivity (0.85) and specificity (0.87). Both psychological (negative affect, certain coping with cancer reactions, lack of sense of control/positive expectations, and difficulties in regulating negative emotions) and biological variables (baseline percentage of neutrophils, thrombocyte count) emerged as important predictors of mental health deterioration in the long run. Personalized break-down profiles revealed the relative impact of specific variables toward successful model predictions for each patient. Identifying key risk factors for mental health deterioration is an essential first step toward prevention. Supervised ML models may guide clinical recommendations toward successful illness adaptation.

## Introduction

The diagnosis and treatment of Breast Cancer (BC) may induce adverse psychological outcomes that may lead to severe anxiety, depression and other mental health symptoms^1,2^. According to the European Society for Medical Oncology (ESMO) clinical practice guidelines on BC and several clinical studies, long-term psychological needs should be addressed toward improving prevention, prognosis and treatment, and supporting patients’ quality of life (QoL) and overall well-being^3^.

Understanding the psychological implications of BC diagnosis and treatment may benefit the self-efficacy of patients toward successful adaptation and promoting psychological resilience^4,5^. Furthermore, it has been shown that enhancing key patient characteristics, such as self-efficacy to cope with cancer, may improve their well-being^6,7^. Thus, the identification of patient characteristics that can predict long-term well-being and adaptation to cancer is critical for early prevention and for enhancing the effectiveness of appropriate psychosocial interventions in caring for BC patients. Overall mental health is considered of primary importance for the course of patient well-being through BC treatments.

To the best of our knowledge, the identification of risk factors for mental health deterioration among diverse sets of patient variables (i.e. psychological traits, life-style, sociodemographic and medical variables) has not been examined prospectively using adaptive learning algorithms in women with BC. The present study focused on a particularly challenging clinical problem, namely the prediction of psychological resilience among patients who did not report significant mental health-related symptoms at the time of diagnosis and are thus less likely to be systematically monitored for signs of mental health deterioration during the course of cancer treatment and thereafter.

We compared patients who displayed adequate psychological resilience throughout the first year post diagnosis, by never reporting increases in symptomatology above a threshold that was suggestive of anxiety and depression, with women who reported poor mental health at the one-year follow up assessment. The first aim of this study was to assess the overall accuracy of a flexible Machine Learning (ML)-based framework for predicting one-year mental health outcomes based on all data registered within the first three months post diagnosis (including mental health and subjective quality of life ratings). A secondary aim was to apply a model-agnostic analysis to aid interpretation of prediction results at the patient level (i.e., identify predictor variables that emerge as key contributors to a given classification result after statistically controlling for all other predictors in the model).

## Results

### Study sample

The initial pool of potential study participants consisted of 600 women who were assessed at baseline (M0) and after three months (M3). Of these, 537 were followed up to twelve months (M12) and met the strict criteria of variable- and case-wise missingness (76.1%) [see Supplementary Information, Fig. S1]. Compared to patients who did not provide useful data at M0, M3 and M12, those who did were older (p = 0.01) and had lower total HADS scores at baseline (p < 0.001) and at M3 (p = 0.05), but not at M12 (p = 0.7). The latter group comprised women who reported higher income (p = 0.001) and education (p = 0.05).

A total of 378 women met the criteria for inclusion into one of the two groups of interest (Stable-Good and Deteriorated Mental Health between M0 and M12): 328 maintained low HADS scores throughout the first year after diagnosis (Stable-Good Mental Health group), while the remaining 50 patients had clinically significant symptomatology at M12 (Deteriorated Mental Health group; see Supplementary Information, Fig. S1). Cases with low HADS scores at M0 and high scores at M12 who displayed substantial fluctuation across the five available measurement waves (e.g., low–high–low–low–high scores or low–low–high–low–high) were not included in this group.

Among the remaining patients, 56 reported reduced symptoms between M0 and M12, 55 women displayed significant symptoms at both M0 and M12, and 48 women could not be classified to one of the four groups (they had missing HADS data on two or more measurement waves or displayed substantial fluctuations on HADS total score across the five waves, e.g., significantly low-significantly high-significantly low or significantly high-significantly low-significantly high).

### Sociodemographic and clinical characteristics of patient groups

Self-reported psychological traits, sociodemographic data, life-style patient characteristics and medical variables are presented in Tables 1, 2, and 3 and described in detail in the Supplementary Information. Overall, patients who reported stable-good mental health had higher education level and income (country-adjusted) and were more likely to be in retirement or self-employed. There were also more likely to engage in at least moderate physical exercise at the time of diagnosis. The two groups were comparable on menopausal status and obesity at the time of diagnosis, presence of cumulative significant life stressors in the period immediately preceding diagnosis, history of anxiety disorder or dysthymia, physical comorbidities and all cancer-related variables registered in the study (i.e., cancer stage, molecular tumor profiles, cancer-related treatments, and frequency of significant medical occurrences during the first three months post diagnosis warranting hospitalization).

**Table 1:** Patient sociodemographic, clinical and life-style characteristics at M0 and M3 measurement waves by mental health change group.

Mental health status (diagnosis to month 12)	Stable-good (n = 328)	Deteriorated (n = 50)	P value
Age (mean (SD) in years)	56.5 (8.1)	54.5 (8.3)	0.1
Education (≥ 9 years)	309 (94.2)	42 (84.0)	0.016
Has children	284 (86.6)	39 (78.0)	0.1
Has partner	246 (74.9)	43 (86.0)	0.1
Currently employed	247 (75.3)	35 (70.7)	0.5
Full-time, retired, or self-employed	293 (89.3)	39 (78.0)	0.035
Unemployed, housewife, or part-time	35 (10.7)	11 (22.2)	
Low income	55 (16.8)	17 (34.1)	0.012
Life stressors (≥ 2)	106 (32.4)	15 (30.0)	0.8
Obesity	63 (19.3)	7 (14.3)	0.5
Family history of BC	112 (34.2)	21 (42.0)	0.3
Physical comorbidity (chronic)	123 (37.5)	17 (34.0)	0.7
Metabolic comorbidity	84 (25.6)	10 (20.0)	0.5
History of anxiety disorder/dysthymia	32 (9.7)	7 (16.0)	0.2
Psychotropic medication	45 (13.8)	9 (18.0)	0.4
Menopausal status			
Premenopausal	96 (29.3)	17 (34.0)	0.6
Perimenopausal	17 (5.2)	2 (4.0)	
Postmenopausal	214 (65.4)	31 (62.0)	
Mental health support by month 3	47 (14.3)	11 (21.7)	0.2
Sick leave (days, mean [SD])	58.8 (81.1)	78.7 (111.8)	0.1
Current smoker	105 (32.1)	15 (30.6)	0.8
Alcohol consumption			
No/occasional	102 (31.2)	16 (32.0)	0.2
Moderate	192 (58.7)	33 (66.0)	
Heavy	33 (10.1)	1 (2.0)	
Diet			
Mediterranean	88 (26.9)	21 (42.0)	0.03
Special diet	52 (16.0)	7 (14.0)	0.8
Exercise			
No/occasional	70 (21.2)	25 (50.0)	< 0.001
Moderate	133 (40.6)	15 (30.0)	
Heavy	125 (38.2)	10 (20.0)	

Counts (%) unless otherwise indicated.

**Table 2:** Cancer-related characteristics at M0 and M3 measurement waves by mental health change group.

Mental health status (diagnosis to month 12)	Stable-good (n = 328)	Deteriorated (n = 50)	P value
Cancer stage			0.4
I	174 (53.0)	24 (47.1)	
II	128 (39.0)	22 (43.1)	
III	26 (8.0)	5 (9.8)	
Molecular tumor characteristics			
Luminal_A	255 (77.6)	34 (68.0)	0.3
Luminal_B	33 (10.0)	6 (12.0)	
Triple_negative	16 (5.0)	6 (12.0)	
HER2_enriched	12 (3.8)	3 (6.0)	
Thrombocytes (× 10^3^/mL, mean [SD])	267.7 (67.4)	244.9 (51.1)	0.027
Creatinine (μmol/L, mean [SD])^1^	66.7 (10.1)	68.5 (12.4)	0.2
Baseline percentage of neutrophils, (mean [SD])	0.60 (0.20)	0.73 (0.21)	< 0.001
Surgery			
Mastectomy	75 (22.7)	15 (30.0)	0.3
Lumpectomy	254 (77.3)	35 (70.0)	0.3
Chemotherapy	167 (50.9)	21 (42.0)	0.3
Adjuvant	121 (36.9)	15 (29.3)	0.3
Neoadjuvant	43 (13.1)	11 (22.2)	
Endocrine therapy	286 (87.2)	39 (78.0)	0.1
Anti HER2 treatment	55 (16.7)	9 (18.0)	0.8
Radiotherapy	273 (83.2)	34 (68.2)	0.02
Hospitalization by month 3	30 (9.2)	5 (10.6)	0.4

Counts (%) unless otherwise indicated.

**Table 3:** Psychosocial characteristics that optimally differentiated patients who displayed stable-good from those who showed deteriorating mental health according to machine learning models 2 and/or 1.

Mental health status (diagnosis to month 12)	Stable-good (n = 328)	Deteriorated (n = 50)	P value
Measured at baseline (M0)			
Manageability (SOC)^1,2^	21.2 (3.5)	18.1 (4.0)	< 0.001
Negative affectivity (PANAS)^1,2^	1.6 (0.5)	1.9 (0.6)	< 0.001
Coping with cancer (CBI)^1,2^	7.5 (0.9)	6.9 (1.2)	< 0.001
Trait resilience^1,2^	3.0 (0.5)	2.7 (0.7)	< 0.001
Forward (PACT)^1,2^	5.4 (0.9)	5.1 (1.0)	0.017
Future perspective^1,2^	60.4 (25.0)	42.7 (30.9)	< 0.001
Optimism (LOT)^1,2^	2.9 (0.6)	2.5 (0.6)	< 0.001
Trauma (PACT)^1,2^	5.4 (0.8)	5.1 (0.8)	0.006
Meaningfulness (SOC)^1^	23.6 (3.3)	21.6 (4.1)	< 0.001
Mindfullness (MAAS)^1,2^	4.5 (0.7)	4.2 (0.8)	0.008
Comprehensibility (SOC)^1^	21.7 (3.6)	19.9 (4.3)	0.002
Arm symptoms (BR-23)^1^	11.1 (15.9)	21.3 (22.0)	< 0.001
Flexibility (PACT)^1,2^	10.2 (1.7)	9.8 (2.0)	0.01
Positive emotion regulation (CERQ)^2^	3.4 (0.7)	3.3 (0.7)	0.1
HADS anxiety^2^	5.2 (2.8)	7.3 (2.5)	< 0.001
HADS depression	2.5 (2.1)	4.4 (2.2)	< 0.001
Global QoL	78.5 (15.6)	69.2 (19.7)	< 0.001
Measured at month 3			
Negative affectivity (PANAS)^1,2^	1.4 (0.5)	2.1 (0.6)	< 0.001
Anxious preoccupation (MAC)^1,2^	1.9 (0.5)	2.4 (0.6)	< 0.001
Helplessness (MAC)^1,2^	1.3 (0.3)	1.6 (0.5)	< 0.001
Social support^1,2^	4.2 (0.8)	3.7 (0.8)	< 0.001
Treatment side effects (BR-23)^1,2^	23.9 (16.4)	30.1 (16.2)	0.01
Avoidance (MAC)^1,2^	2.3 (0.7)	2.7 (0.6)	< 0.001
Body image (BR-23)^2^	80.3 (20.7)	70.7 (22.6)	< 0.001
Community cohesion (FARE)^1^	6.2 (0.9)	6.0 (0.9)	0.2
Emotional support^1,2^	4.2 (0.8)	3.8 (0.8)	< 0.001
Future perspective^1,2^	65.6 (20.6)	49.9 (27.1)	< 0.001
Positive affectivity (PANAS)^1,2^	3.45 (0.6)	3.3 (0.6)	0.04
PTGI^2^	2.4 (1.2)	2.8 (1.1)	0.027
HADS anxiety^2^	4.1 (2.6)	7.7 (2.7)	< 0.001
HADS depression^2^	2.9 (2.6)	7.3 (3.2)	< 0.001
Global QoL^2^	72.8 (18.9)	62.1 (17.3)	< 0.001

Values are means (SD). Superscripts indicate important features within Model 1 and 2, respectively. *SOC* Sense of coherence, *LOT* Life orientation test, *MAAS* Mindful attention awareness scale, *PANAS* Positive and negative affect, *CERQ* Cognitive emotion regulation questionnaire, *CBI* Cancer behavior inventory, *PACT* Perceived ability to cope with trauma, *BR-23* Breast cancer-23, *MAC* Mental adjustment to cancer, *PTGI* Post-traumatic growth inventory, *FARE* Family resilience, *HADS* Hospital Anxiety and Depression Scale.

### Optimized prediction of one-year adverse mental health outcomes

As shown in Table 4, Model 1 (which considered all available data collected at M0 and M3 including mental health and subjective quality of life ratings) correctly predicted one-year mental health deterioration for 85% of patients. Moreover, the model identified the patients who had stable good mental health status at M12 with approximately 87% certainty. The shape of the Receiver Operating Characteristic Curve shown in Supplementary Information, Fig. S2 (AUC = 0.864) illustrates a fair balance between sensitivity and specificity.

**Table 4:** Performance of the two ML models in predicting one-year mental health deterioration as outcome (mean ± SD).

Performance metrics	Model 1	Model 2
Specificity	0.866 ± 0.01	0.759 ± 0.0
Sensitivity	0.846 ± 0.01	0.747 ± 0.0
Accuracy	0.790 ± 0.0	0.747 ± 0.03
Precision	0.845 ± 0.0	0.750 ± 0.0
F1	0.542 ± 0.0	0.401 ± 0.01
AUC	0.864 ± 0.0	0.790 ± 0.0

Models 1 and 2 are identical with the exception that Model 1 includes HADS Anxiety, HADS Depression, and Global Quality of Life ratings at M0 and M3 as predictors.

**Figure 1:**
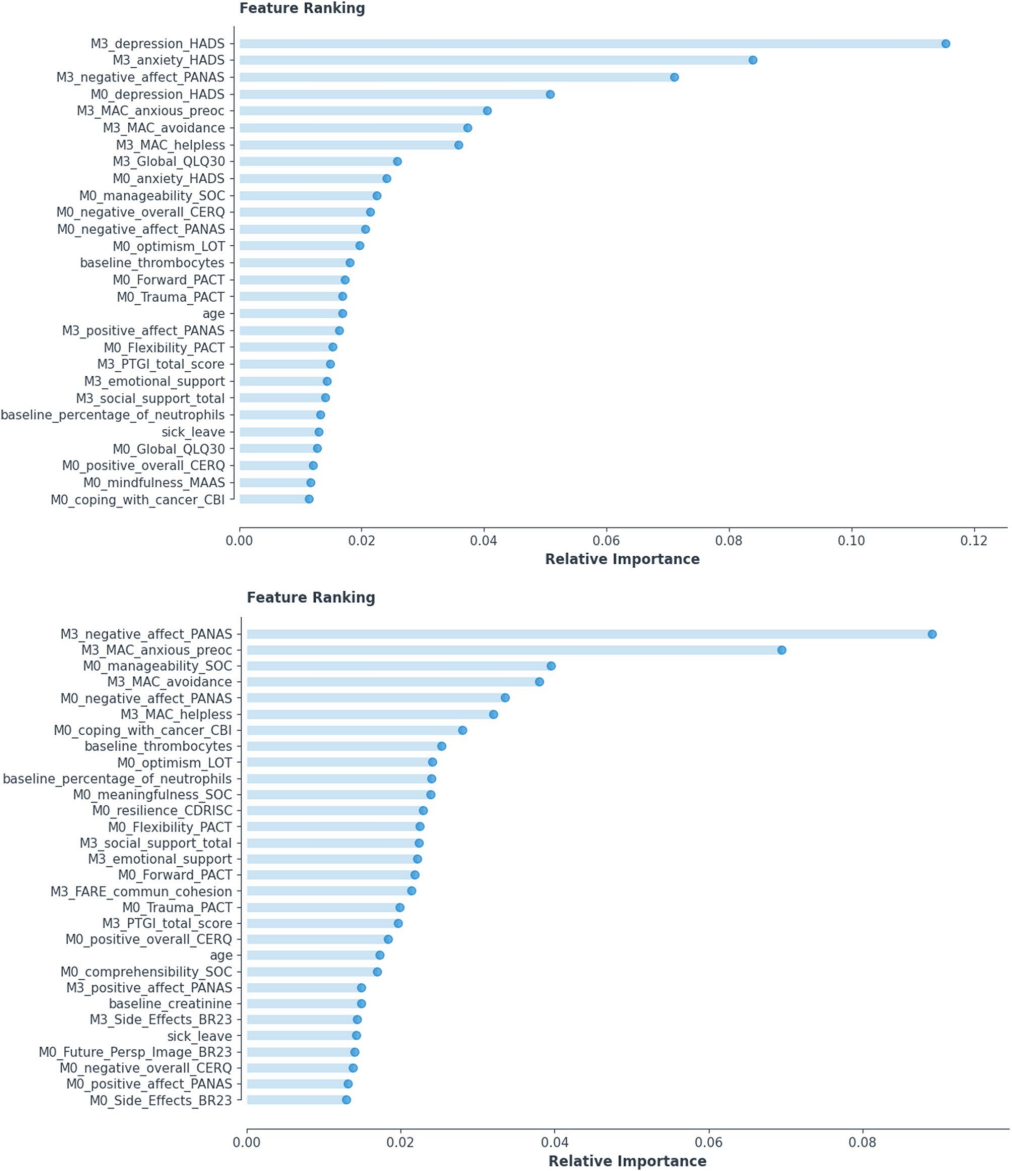
The selected features for Model 1 (upper panel) and Model 2 (lower panel) ranked according to their relative importance for prediction of mental health change between M0 and M12. M0 indicates variables assessed at baseline and M3 variables assessed at M3.

Most important predictors included variables measured shortly after disease diagnosis, as well as variables reported at the 3-month follow-up (that is, during treatment; see Fig. 1, upper panel). They comprised general medical-related variables, trait resilience and other psychological characteristics presumed to be associated with illness adaptation, emotional status of the patient (particularly on month 3), and specific illness-related physical symptoms. In addition, two biological variables ranked among the important predictors: thrombocyte count and baseline percentage of neutrophils. Descriptive statistics of the selected continuous variables are shown in Table 3, whereas group data on exercise at M0, which also emerged as an important predictor, is shown in Table 1. As expected, the Stable Mental health group reported significantly lower symptomatology and better global QoL at both M0 and M3 (p < 0.001). Additional variables which emerged as important predictors are listed in Table 3.

Results were compared with the output of a reference model using logistic regression. Variables were force-entered into this model if their association with the dependent (categorical) variable approached significance (p < 0.1). In total, 38 variables met this criterion, and the model represented a good fit to the data, X^2^ (38) = 133.75, p < 0.001, R^2^ = 0.599. The logistic function achieved very high specificity (97%) and considerably lower sensitivity (56%).

### Personalized risk profiles

Model 2 (which did not consider mental health and subjective quality of life ratings at M0 and M3) correctly predicted one-year mental health deterioration for 75% of patients (see Table 4). Moreover, the model identified patients who had stable good mental health status at 12 with approximately 76% certainty. The shape of the ROC curve shown in Supplementary Information [Fig. S3] (AUC = 0.79) illustrates a fair balance between sensitivity and specificity.

### Identification of modifiable predictors of mental health status

As shown in Fig. 1 (lower panel), important predictors of 12-month patient’s mental health status in the total sample (without considering mental health and QoL indices at M0 and M3) included variables measured shortly after disease diagnosis as well as variables reported at the three-month follow-up (that is, during treatment). Overall emotional state (negative affect) measured at baseline and M3, certain coping reactions at M3 (i.e., anxiety preoccupation, avoidance, and helplessness), a sense of control over adversities (i.e. sense of coherence) and baseline percentage of neutrophils featured strongly among the most highly ranked predictors of mental health deterioration. In addition, certain other variables emerged as significant predictors of mental health, such as, social support, self-efficacy to cope with cancer, resilience as trait, the ability to cope with trauma, family cohesion, future perspective, optimism, and some specific symptoms (e.g., side-effects), which might also be considered as potential targets of appropriate clinical interventions. Importantly, the two biological variables highlighted by Model 1 also featured in the list of important predictors for Model 2 (thrombocyte count, baseline percentage of neutrophils) in addition to serum creatinine levels measured at baseline.

**Figure 2:**
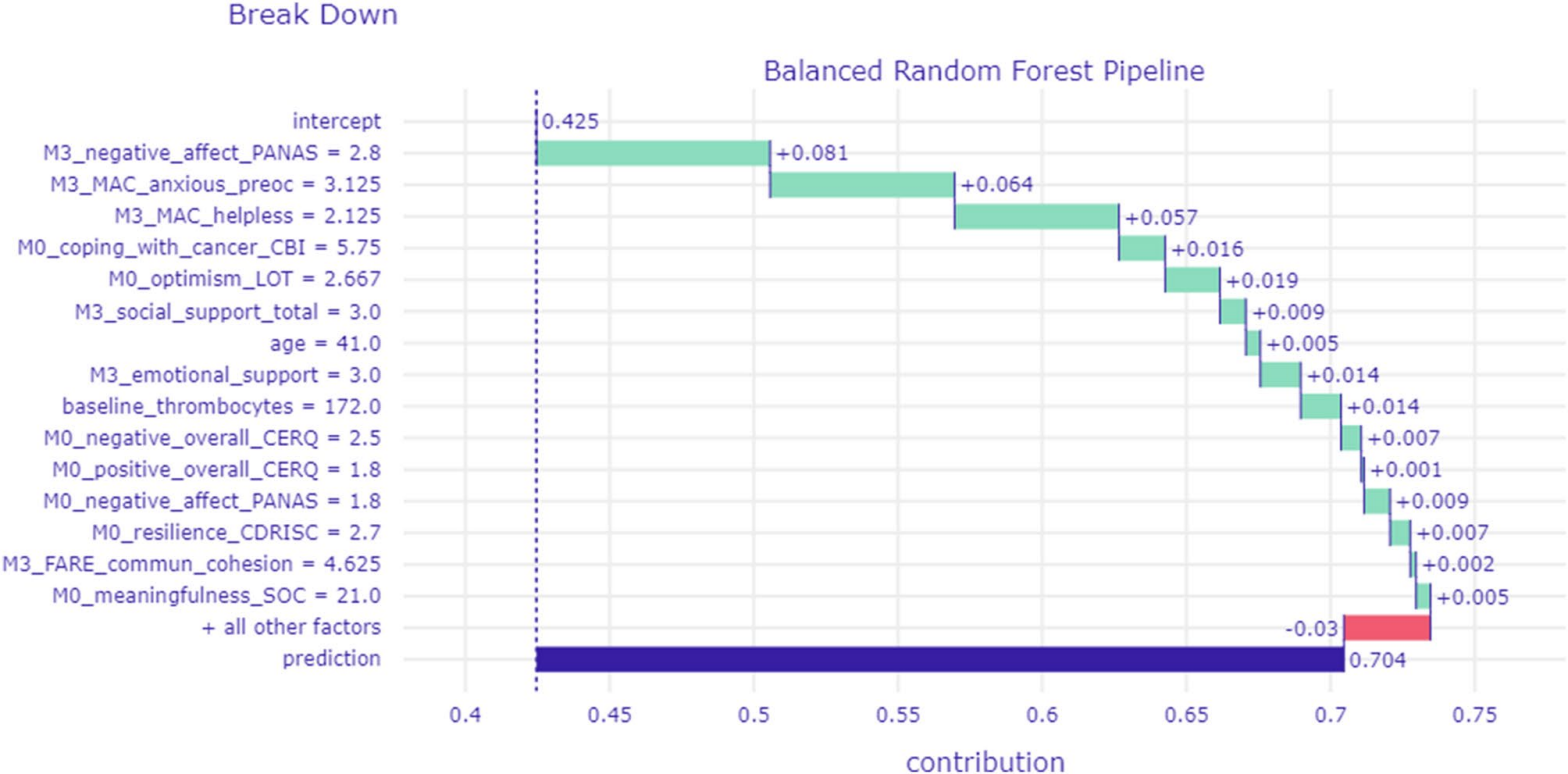
BD profile of a patient who displayed deteriorated mental health status at M12. Prediction probability is shown on the horizontal axis (Stable-Good mental health = 0, Deteriorated Mental Health = 1). This patient was given a high probability of membership in the Deteriorated Mental Health class (0.704). Actual patient scores on each predictor variable are shown on the left-hand side. A positive value assigned to a given score (green bars) indicates the degree of its contribution toward a prediction of Deteriorated Mental Health. A negative value (red bars) indicates the degree of a given score’s contribution away from a prediction of Deteriorated Mental Health (i.e., increasing the probability of assigning this patient to the Stable-Good Mental Health class).

The BD plot presented in Fig. 2 illustrates the contribution of a subset of variables toward a correct prediction of Deteriorated Mental Health for a randomly selected participant, as indicated by an estimated probability of 0.704 to belong in Class 1 (deteriorated mental health vs Class 0 [stable mental health]). The 15 most highly ranked features selected by the RF model for this specific instance-level prediction are displayed for demonstration purposes. All variables seem to “facilitate” the adverse mental health outcome for this patient as indicated by positive weights (shown in green). This effect can be accounted for by notably high scores on certain variables in the upper quartile of the sample distribution (i.e., Negative Affectivity, anxious preoccupation, and helplessness at M3). In addition, prediction of mental health decline for this patient is explained by very low scores (in the lower quartile of the sample distribution) on characteristics that are presumed to protect against poor mental health outcomes, namely cancer coping strategies and optimism. Finally, a relatively low platelet count at the time of diagnosis may further predispose this patient to the development of emotional difficulties.

**Figure 3:**
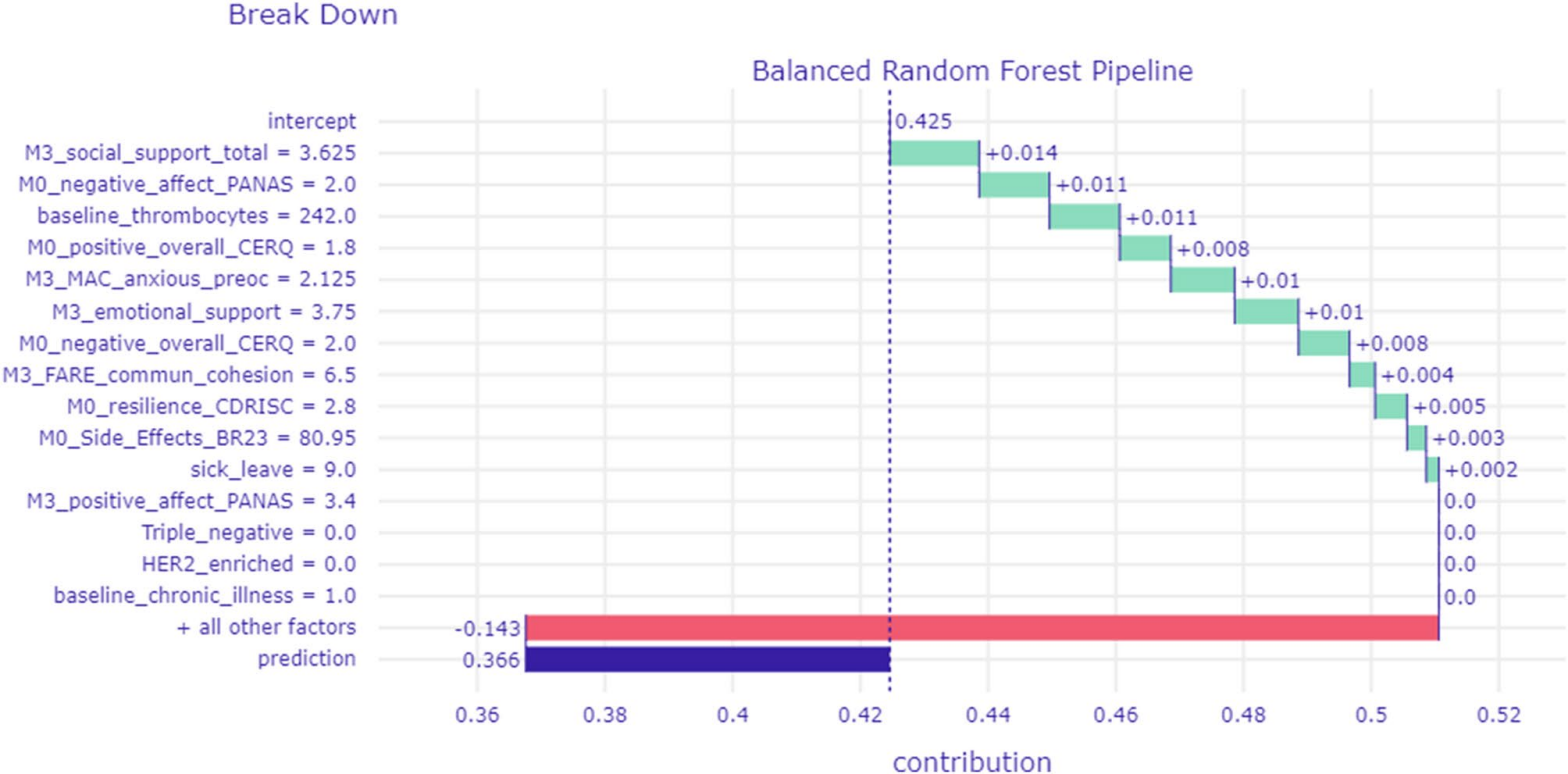
BD profile of a patient who displayed stable good mental health status as indicated by a very low probability of membership in the Deteriorated Mental Health class (0.366). All other figure elements are the same as in Fig. 2.

Figure 3 illustrates the BD profile for a patient who was correctly predicted to have displayed stable-good mental health status over time, as indicated by an estimated probability of 0.366 to belong in Class 1. The BD profiles of these patients reveal a much more complex picture. This patient reported average levels of negative affectivity and anxious preoccupation and also average scores on trait resilience and sense of community cohesion. She also reported relatively low levels of social and emotional support. Interestingly, all these scores increased the probability of an adverse mental health outcome which, nevertheless, remained low. It appears that certain patient characteristics may represent underlying psychosocial vulnerabilities which were effectively counteracted by the combination of all other sociodemographic, biological, life-style, and clinical characteristics (as indicated by the notable negative weight of “all other factors” toward reducing the probability of an adverse mental health outcome [shown in red]).

## Discussion

The present study used adaptive learning algorithms to address a challenging clinical problem in cancer care: predicting adverse mental health outcomes among patients who manifest fairly good initial emotional response to the diagnosis and the prospect of cancer treatments. Specifically, we sought to identify patients who displayed lower resilience and clinically significant deterioration in mental health one year later from patients who displayed stable-good mental health. Supervised learning analysis was conducted on a large, heterogenous set of variables obtained in the context of a prospective, multinational study on the predictors of psychosocial resilience following diagnosis of BC. The design of the present analyses was guided primarily by the potential clinical utility of the results. Thus, the supervised learning models considered variables that could be readily available to clinicians in future practice, namely medical, sociodemographic, and life-style variables, integrated with a select set of psychosocial patient characteristics. To optimize prediction accuracy, we implemented an ML model that took into account the most proximal variables to the targeted clinical outcome, namely mental health and subjective QoL ratings collected during the first three months post-diagnosis (Model 1). In a supplementary model (Model 2) we excluded these variables in favor of psychological characteristics and behaviors to estimate personalized risk profiles. The latter included several potentially modifiable traits which can serve as targets of customized psychological interventions.

A rigorous analytic approach was employed to mitigate some of the commonly observed pitfalls of machine learning approaches, namely overfitting and poor model generalizability. To address these issues our pipeline entailed feature selection, model training, validation and testing based on the four clinical sites that contributed to the BOUNCE prospective study. Further, the generalizability of the prediction model was assessed on cases that were not considered during the training phase, to avoid overfitting and reduce classification errors. The prediction model correctly classified 85% of the cases that displayed clinically significant mental health change at M12. Moreover, the model identified the patients who had stable-good mental health status at M12 with approximately 86% certainty. Hence, our results inspire optimism on transferring our prediction model to relevant clinical settings.

We employed patient-level interpretation techniques to identify key variables that contribute to the successful predictions for individual patients (Figs. 2, 3). Pending future validation, these results reflect the impact of each predictor variable to the estimated probability that a given patient is assigned to the low- or high-risk class (i.e., Stable-Good and Deteriorated Mental Health groups, respectively). In principle, these indices of relative variable impact could help clinicians identify patient characteristics which may predispose toward, or have a protective role against, adverse mental health outcomes.

The variables that emerged as significant predictors of changes in mental health symptoms over one year (i.e. stable-good or deterioration at M12) correspond to certain “clusters” of factors, namely: (a) negative affect; (b) coping with cancer responses and self-efficacy to cope with cancer; (c) a sense of control/positive expectations (i.e., sense of coherence; optimism); (d) social and family support; (e) certain life-style factors (i.e., exercise) and, (f) certain treatment-related symptoms (e.g., arm symptoms). These findings are in accordance with the major psychological theories on adaptation to severe illness, including BC, such as the Common Sense Model^8^ or the Transactional Stress Model^9^. In this framework, adaptation to a severe health crisis is a complex process which is determined by: (a) a variety of personal and interpersonal resources, such as expectations, life-style, or social support, which may buffer the negative impact of the situation and facilitate adaptation; (b) cognitive-emotional processes, such as affect and emotion regulation and self-efficacy to cope with cancer, that guide behaviors such as preoccupation and helplessness; (c) contextual and specific stressor-related factors that may impact adaptation directly or indirectly, such as physical symptoms. In addition, the findings pinpoint those early factors, coming from a large array of sociopsychological, medical, and life-style variables, that are significant predictors of the present study outcome and, in this way, will guide the efforts to develop appropriate clinical recommendations which may guide informed and shared decision making between health professionals and patients, to promote adapted mental health status.

Finally, it is worth noting that few medical variables emerged as significant predictors of the M12 overall mental health change. These included specific symptoms related to BC treatments^10,11^, indices of immune response, and relatively higher thrombocyte count^12,13^. The role of BC treatments or subsequent mental and functional well-being has been highlighted by recent studies^10,11^ however in our study treatments were not among the strong predictors. At present we may only surmise on the mechanisms responsible for the impact of biological measures. Baseline percentage of neutrophils, which has been highlighted as an index of systemic inflammation^14^, may be both directly and indirectly involved in the pathways leading to increased mental health symptomatology. Thus, systemic inflammation may impact cognitive functions, as recently shown among cancer patients^15^, impacting the patient’s capacity to cope with everyday tasks; and also indirectly by reducing her capacity to engage cognitive resources required for active coping (such as for engaging in cognitive reappraisal). The clinical relevance of thrombocyte count and creatinine in our study remains unclear and should be interpreted with caution as mean values were within normal limits in both groups.

Other medical factors (such as cancer stage and histological tumor characteristics) may exert their influence on mental health and QoL indirectly; that is, through the cognitive-emotional, behavioral, and situation specific variables described above. Future analyses will examine the potential indirect impact of medical factors on the outcomes. As more measurement waves become available from the BOUNCE study, advanced statistical and computational models can be applied to explore the complex interplay of treatments side effects, life events, emotional, behavioral, and cognitive processes over time.

The findings of this study should be considered in light of certain limitations. The most significant concerns the use of a patient-reported clinical outcome. Although HADS is a very reliable and widely used scale, it still reflects only patients’ perception of their condition. Moreover, the set of variables selected by the ML pipeline as important predictors of mental health deterioration depend to some extent upon the characteristics of the comparison group, which included only those patients who exhibited consistently high levels of psychological resilience—selected to optimize group separation (and consequently model performance).

Finally, generalizability was examined with the use of subsamples randomly selected from the same patient cohort. Future studies should also use external samples of patients to assess the generalizability of the models. Although a very large number of variables was included in the model, still not everything could be assessed. For example, the impact of factors related to the health care system or the delivery of health services that are particular in each participant country, was not examined. In this regard, future similar studies could focus on such variables.

Despite these limitations, the practical implications of the findings of this study are considerable. The identification of a few major predictors may help towards the early recognition of patients who are at higher risk for a deteriorated psychological health. A systematic assessment of these predictors may prove to be crucial for the prevention of psychological difficulties in the long run. Moreover, the identification of individual patient characteristics that contribute to long-term mental health deterioration, through the development of personalized break-down profiles, may facilitate the development of personalized psychological support programs that may meet each patient’s needs more effectively and thus achieve a better outcome.

## Conclusions

We developed and implemented a supervised learning analysis pipeline to model resilience, in terms of change in mental health status between M0 and M12 measurement waves. We sought to identify those predictors that could discriminate between patients who displayed stable-good mental health vs. those who showed clinically significant deterioration in mental health at one-year post diagnosis. According to our findings, a small set of cognitive/emotional factors and personal characteristics can best predict adaptation to an adverse health event and, as a result, mental health status during the illness progression. These factors could become the focus of systematic interventions to refine clinical recommendations while maximizing patient management and care.

## Methods

### Study population

The BOUNCE study took place in four European countries (Finland, Italy, Israel and Portugal) aiming to evaluate psychosocial resilience of BC patients during the first 18 months post-diagnosis as a function of psychological (trait and ongoing), sociodemographic, life-style, and medical variables (disease and treatment-related) (H2020 EU project BOUNCE GA no. 777167; for more information see https://www.bounce-project.eu/). The study enrolled 706 women between March 2018 and December 2019 according to the following criteria: (i) Inclusion: age 40–70 years, histologically confirmed BC stage I, II, or III, surgery as part of the treatment, some type of systemic therapy for BC; (ii) Exclusion: History or active severe psychiatric disorder (Major depression, bipolar disorder, psychosis), distant metastases, history or treatment of other malignancy within last 5 years, other serious concomitant diseases diagnosed within the last 12 months, major surgery for a severe disease or trauma within 4 weeks prior to study entry, or lack of complete recovery from the effects of surgery, pregnancy or breast feeding. The BOUNCE study is a longitudinal, observational study involving seven measurement waves: Baseline (taking place 2–5 weeks after surgery or biopsy and considered as Month 0 [M0], and subsequently at three-month intervals (M3, M6, M9, M12, M15, M12) with a final follow up measurement at M18. Data on each of the main outcome variables were collected at all time points. Data from the remaining time points served secondary research goals of the overall project.

The entire BOUNCE study was approved by the ethical committee of the European Institute of Oncology (Approval No R868/18—IEO 916) and the ethical committees of each participating clinical center. All participants were informed in detail regarding the aim and procedural details of the study and provided written consent. All methods were carried out in accordance with relevant guidelines and regulations.

### Predictor variables

For the current analyses we considered sociodemographic, life-style, medical variables and self-reported psychological characteristics registered at the time of BC diagnosis and, also, at the first follow up assessment, conducted 3 months after diagnosis. The decision to pool predictor data from the first three months post diagnosis was guided by the following considerations: (a) Emotional responses and awareness of emotional and behavioral adaptive processes are often not fully developed until the full scope of the illness can be appreciated by the patient, (b) This period defines a realistically short observation window to record resilience predictors in routine clinical practice, yet not too long in view of the one-year study end-point, (c) previous studies have shown that significant changes in psychological well-being typically take place later in the trajectory of illness.

### Outcome variable

Self-reported mental health status at 12 months post-diagnosis, indexed by the total score on the 14-item Hospital Anxiety and Depression Scale (HADS)^16^, served as the outcome variable in the current analyses (see Supplementary Information). The clinically validated cutoff score of 16/42 points in a wide range of languages was used to identify patients who reported potentially clinically significant symptoms at M0 and at M12^17,18^. Subsequently, patients were assigned to two classes: (a) those who reported non-clinically significant symptoms of anxiety and depression at M0 (i.e., immediately following BC diagnosis) and clinically significant symptomatology at M12 (i.e., one year post diagnosis) according to validated cutoffs on HADS total score (Deteriorated Mental Health group), and (b) those who reported mild symptomatology throughout the first year post diagnosis (Stable-Good Mental Health group). Thus, the Deteriorated Mental Health group comprised persons who scored < 16 points at M0 and ≥ 16 points at M12, whereas the Stable-Good Mental Health group comprised persons who scored < 16 points at M0, M3, M6, M9 and M12 assessment time points.

### Data analyses

The analysis pipeline adopted to address the main and secondary objective of the study entailed preprocessing steps, feature selection, model training and testing^19^. Model 1 was designed to optimize prediction of one-year adverse mental health outcomes by considering all available variables collected at M0 and M3, including HADS Anxiety, HADS Depression, and Global QoL. Model 2 was designed to obtain personalized risk profiles and focus on potential modifiable factors (by omitting HADS Anxiety, HADS Depression, and Global QoL measured at M0 and M3). Feature selection, using a Random Forest algorithm, was incorporated into the ML-based pipeline alongside the classification algorithm to select only the relevant features for training and testing the final model (see Supplementary Information). The area under the Receiver Operating Characteristic curve (ROC AUC) was used to evaluate the performance of the cross-validated model on the test set by estimating the following metrics: specificity, sensitivity, accuracy, precision, F-measure, and AUC.

### Data preprocessing and handling of missing data

Initially, raw data were rescaled to zero mean and unit variance and ordinal variables were recoded into dummy binary variables. Cases and variables with more than 90% of missingness were excluded from the final dataset. Remaining missing values were replaced by the global median value (supplementary analyses showed that applying multivariate imputation had negligible effect on model performance; see Supplementary Material).

### Feature selection

Feature selection was conducted using a meta-transformer built on a Random Forest (RF) algorithm^20^ which assigns weights to the features and ranks them according to their relative importance. The maximum number of features to be selected by the estimator was set to the default value (i.e. the square root of the total number of features) in order to identify all important variables that contribute to the risk prediction of mental health deterioration. The feature selection scheme was incorporated into the ML-based pipeline alongside the classification algorithm to select only the relevant features for training and testing the final model.

### Model training and validation

To address the rather common problem of model overfitting in machine learning applications in clinical research we adopted a cross-validation scheme with holdout data for the final model evaluation. Model overfitting occurs because a model that has less training error (i.e. misclassifications on training data) can have poor generalization (expected classification errors on new unseen data) than a model with higher training error. As a result, we took extra steps to avoid partially overlapping subsets of cases by splitting our dataset into training and testing subsets with a validation set. Hence, model testing was always performed on unseen cases which were not considered during the training phase and, consequently, did not influence the feature selection process. This procedure helps to minimize misclassifications on the training phase while also ensuring lessening of generalization errors.

In the present study, a fivefold data split for hyper-parameters (i.e. cross-validation with grid search) was applied on the training, testing and validation subsets, to prevent overfitting and maximize model generalizability performance on the test set. A grid search procedure with an inner fivefold cross-validation was applied on the validation set for hyper-parameters tuning and model selection. To this end, the best parameters from a grid of parameter values on the trained models were selected enabling the optimization of the classification results on the test set.

### Classification with balanced random forest algorithm

Class imbalance handling was addressed using random under-sampling methods to balance the subsets combined inside an ensemble. Specifically, a balanced random forest classifier from the imbalanced-learn MIT-licensed library^21^ was applied to deal with the classification of imbalanced classes within our dataset. Balanced Random Forest^22^ combines the down sampling majority class technique and the ensemble learning approach, artificially adjusting the class distribution so that classes are represented equally in each tree in the forest. In this manner, each bootstrap sample contains balanced down-sampled data. Applying random-under sampling to balance the different bootstraps in an RF classifier could have classification performance superior to most of the existing conventional ML-based estimators while alleviating the problem of learning from imbalanced datasets.

The following metrics to assess the performance of the learning algorithm applied on imbalanced data: specificity (true negative rate); sensitivity (true positive rate); accuracy, precision, and F-measure. These metrics are functions of the confusion matrix given the (correct) target values and the estimated targets as returned by the classifier during the testing phase. We also used the Receiver Operating Characteristic (ROC) curve to represent the tradeoff between the false negative and false positive rates for every possible cut off. The Area Under the Curve (AUC) was also computed according to the estimated ROC analysis.

### Personalized risk profiles (model 2 only)

Following the analysis steps described in the preceding paragraphs, model-agnostic analysis was implemented on the set of variables that emerged as significant features from Model 2 to identify predictor variables of primary importance for a particular mental health prediction^23,24^. This analysis supports the interpretability of the set of variables that emerged as significant features toward patient classifications. Specifically, model agnostic analysis can be applied: (i) at the global (variable-specific) level to help clarify how each feature contributes toward model decisions per patient group and, (ii) at the local (i.e., patient-specific) level to identify predictor variables of primary importance for a particular mental health prediction. In view of the lack of precedence in the literature we selected mathematical models that made no assumptions about data structure. The break-down plots (local level) were developed using the *dalex* Python package^19,23^ with the default values in the arguments of the main function were applied.

## Supplementary Information

Supplementary Figures.

## Data Availability

The data that support the findings of this study are available from BOUNCE consortium, but restrictions apply to the availability of these data, which were used under license for the current study, and so are not publicly available. Data are however available from the authors upon reasonable request and with permission of BOUNCE consortium.
