# Supplementary material for: Personalized prediction of one-year mental health deterioration using adaptive learning algorithms: a multicenter breast cancer prospective study
Source: Sci Rep. 2023 Apr 29;13:7059. doi: 10.1038/s41598-023-33281-1 (PMC10148884; doi:10.1038/s41598-023-33281-1)
Supplement: Supplementary file 1 — Supplementary Figures. [file 41598_2023_33281_MOESM1_ESM.pdf]

# Personalized prediction of one-year mental health deterioration using adaptive learning algorithms: A multicenter breast cancer prospective study

[1,2]Konstantina Kourou, [3]Georgios Manikis, [1,2]Eugenia Mylona, [4]Paula Poikonen-Saksela, [5,6]Ketti Mazzocco, [7]Ruth Pat-Horenczyk, [8,9]Berta Sousa, [9,10]Albino J. Oliveira-Maia, [4]Johanna Mattson, [11]Ilan Roziner, [5]Greta Pettini, [3]Haridimos Kondylakis, [3]Kostas Marias, [12]Mikko Nuutinen, [3,13]Evangelos Karademas, [3,14]Panagiotis Simos and [1,2]Dimitrios I. Fotiadis*

[1]Unit of Medical Technology and Intelligent Information Systems, Dept. of Materials Science and Engineering, University of Ioannina, Ioannina, Greece

[2]Foundation for Research and Technology-Hellas, Biomedical Research Institute, Ioannina, Greece

[3]Foundation for Research and Technology-Hellas, Institute of Computer Science, Heraklion, Greece

[4]Helsinki University Hospital Comprehensive Cancer Center and Helsinki University, Finland

[5]Applied Research Division for Cognitive and Psychological Science, European Institute of Oncology IRCCS, Milan, Italy

[6]Dept. of Oncology and Hemato-oncology, University of Milan

[7]School of Social Work and Social Welfare,The Hebrew University of Jerusalem, Israel

[8]Breast Unit, Champalimaud Clinical Centre, Champalimaud Foundation, Lisbon, Portugal

[9]Champalimaud Research and Clinical Centre, Champalimaud Foundation, Lisbon, Portugal

[10]NOVA Medical School, NMS, Universidade Nova de Lisboa, Lisbon, Portugal

[11]Dept. of Communication Disorders, Sackler Faculty of Medicine, Tel Aviv University, Israel

[12]Nordic Healthcare Group, Helsinki, Finland

[13]Dept. of Psychology, University of Crete, Rethymno, Greece

[14]School of Medicine, University of Crete, Heraklion, Greece

***indicates corresponding author (fotiadis@uoi.gr)**

# Supplementary Information

## *Measures*

### *Outcome variable and patient grouping*

HADS employs a 4-point Likert scale to assess frequency of anxiety- and depression-related symptoms with acceptable internal consistency (Cronbach's $\alpha = 0.90$). Higher scores indicate more frequent psychological symptoms.

### *Predictors*

*Sociodemographic.* The following variables were registered at baseline: Age (in years), education level (categorized as low [0-9 years] and high [>9 years]), relational status (alone, or with partner), children (yes, no), employment status (currently employed or not), type of employment (full-time, retired, or self-employed vs. unemployed, housewife, or part-time employment), monthly income (very low vs average/high; adjusted for the Gross Domestic Product of home country of each participant). Two additional variables were aggregated over the first 3 months post diagnosis: sick leave taken (in days), and significant life stressors (other than BC-related) during the first three months post diagnosis (categorized as none/single event vs two or more events).

*Life-style.* The following variables were registered at baseline: Current smoker, alcohol consumption (no drinking or occasional consumption, defined as: ≤2 servings of beer and/or ≤1 servings of spirits per week, moderate, defined as: 3-6 servings of beer and/or ≤4 servings of spirits per week, heavy, defined as: >6 servings of beer and/or >4 servings of spirits per week), self-defined diet (Mediterranean, special diet [e.g., vegan, lactose-free), undefined), physical exercise (defined as: no/occasional [<60 min/week], moderate [60-180 min/week], heavy [>180 min/week]).

*Medical.* Health-related variables collected at baseline: Eastern Cooperative Oncology Group (ECOG) performance status, obesity, family history of BC, pre-existing chronic physical illness (other than metabolic), psychotropic medications (including sleep medications), pre-existing metabolic disease, pre-

existing anxiety or dysthymia, anemia, menopausal status (premenopausal, perimenopausal, postmenopausal), serum levels of alanine aminotransferase, creatinine, and bilirubin, blood cell count (thrombocyte count, baseline percentage of neutrophils).

*Breast cancer-related:* cancer stage (I vs II or III), tumor molecular profile (Luminal A, Luminal B, Triple Negative, HER2 Enriched), progesterone receptor positivity, estrogen receptor positivity, HER2 positivity, Ki67 levels (≥25); treatment-related: surgery at M0, surgery at M3, onset of chemotherapy at M0, onset of chemotherapy at M3, onset of radiotherapy at M0, onset of radiotherapy at M3, type of breast surgery (lumpectomy vs mastectomy), type of chemotherapy (adjuvant or neoadjuvant), type of endocrine therapy (letrozole, exemestane, anastrozole, ovarian suppression, tamoxifen), anti-HER2 therapy, systematic mental health support through M3.

Finally, patient *psychosocial* characteristics were assessed using standardized questionnaires that had been appropriately adapted and translated into the different languages of the four clinical sites of the BOUNCE prospective study. The following domains were assessed: (i) several personality characteristics, (ii) coping and the ability to cope, (iii) perceived social support, (iv) resilience as trait, (v) illness perception and related behaviors, (vi) global QoL and (vii) patient affect at the time of measurement (detailed presentation of these measures can be found in the Supplementary Material).

***Psychosocial measures***

Positive and Negative affect. The Positive and Negative Affectivity Schedule (PANAS) [3] was used to evaluate positive (10 adjectives; Cronbach's $\alpha = 0.84$) and negative affect (10 adjectives; Cronbach's $\alpha = 0.75$). A 5-point Likert type scale was adopted to assess affect over the past week. Higher scores represent higher levels of positive and negative affect, respectively.

Fear of Cancer Recurrence Inventory. The 9-item Fear of Cancer Recurrence Inventory (FCRI) questionnaire was used to measure the fear of a recurrence event [4]. Each item of the questionnaire is rated on a Likert type scale ranging from 0 ("not at all" or "never") to 4 ("a great deal" or "all the time"). The

total score can be obtained by summing the responses to all items. Higher scores indicate higher levels of FCR.

Quality of Life. Global health-related Quality of Life (QoL) was assessed using the corresponding item from the European Organization for Research and Treatment of Cancer (EORTC) QLQ-C30[5]. Specific aspects of QoL were also assessed using this 30-item questionnaire (physical, role, emotional, cognitive and social functioning, fatigue, pain, nausea and vomiting, dyspnea, insomnia, appetite loss, constipation, diarrhea and financial difficulties). Additional, cancer-specific aspects of health status were assessed using the BR-23 module of the EORTC questionnaire. This module comprises of 23 questions related to the (i) disease symptoms, (ii) side effects of treatment (surgery, chemotherapy, radiotherapy and hormonal treatment), (iii) body image, (iv) sexual functioning and (v) future perspective. A linear transformation was applied to the raw scores to reach a range from 0 to 100.

Illness perception and coping responses. The brief version of the Cancer Behavior Inventory (CBI-B) measure [6] was used to assess a general sense of perceived self-efficacy to cope with the illness-related difficulties and needs. A single score measure of coping self-efficacy was yielded (Cronbach's $\alpha = 0.89$) with higher scores indicating higher confidence in coping with illness. The Mental Adjustment to Cancer scale (MAC) [7] was used to estimate patients' coping responses to cancer. The scale includes five reliable dimensions: (i) fighting spirit, (ii) helplessness, (iii) anxious preoccupation, and (iv) avoidance. A 4-point Likert type scale indicate the coping responses of BC patients. Also, the Perceived Ability to Cope with Trauma (PACT) questionnaire was used to estimate the flexibility in coping across different potentially traumatic events [8]. Two scales are measured related to: (i) the focus on processing the trauma (trauma focus), and (ii) the focus on moving beyond the trauma (forward focus). An overall PACT flexibility score was created to evaluate both types of coping. Finally, to assess any potential positive responses to the entire stressful experience, we used the total score on the 14-Post-Traumatic Growth Inventory (PTGI short form) questionnaire (with higher scores indicating better posttraumatic growth) [9].

Social support and family resilience. The modified Medical Outcomes Study Social Support Survey (mMOS-SS) was used to assess social support, which has been shown to provide many benefits related to

overall health and well-being [10]. It consists of 8 items and the total score was calculated by summing all response values (Cronbach's $\alpha = 0.92$). Higher total and subscale mMOS-SS scores reflect stronger social support. For the assessment of family resilience the Walsh Family Resilience Questionnaire [11] was used. For the purposes of the BOUNCE study, two subscales were used: (i) communication and cohesion and (ii) perceived family coping. A higher total score indicates higher levels of family resilience.

Resilience as a personality characteristic (trait). The Connor-Davidson Resilience Scale was used to assess resilience as a trait (CD-RISC) [12]. The scale includes 10 items for quantifying the level of self-perceived resilience (e.g. ability to adapt to change; achieving my goals). Each item is rated on a 5-point Likert type scale from 0 ("not true at all") to 4 ("true nearly all the time") with higher total scores reflecting higher resilience levels (Cronbach's $\alpha = 0.89$).

Emotion regulation and relevant strategies. The Cognitive Emotion Regulation Questionnaire (CERQ – short) was used to identify the cognitive emotion regulation strategies (or cognitive coping strategies) that BC patients followed when experiencing negative events or situations [13]. A 5-item Likert type scale was used for each item ranging from 1 ("(almost) never") to 5 ("(almost) always"). In addition, the Mindful Attention Awareness Scale (MAAS) [14] was used to assess the patients' characteristic of mindfulness. A total score is considered by summing all patients' responses with higher scores reflecting higher levels of dispositional mindfulness.

Other personality characteristics. Sense of coherence was assessed based on the Sense of Coherence (SOC)-13 questionnaire (Cronbach's $\alpha = 0.81$, for the total score). Comprehensibility (5 items), manageability (4 items), and meaningfulness (4 items) were measured on a 7-point (Likert-type) response scale (from 1 (lower) to 7 (higher)) with higher total scores indicating higher level of sense of coherence. Generalized optimism was assessed with the Life Orientation Test (LOT)–Revised (Cronbach's $\alpha = 0.71$)[15].

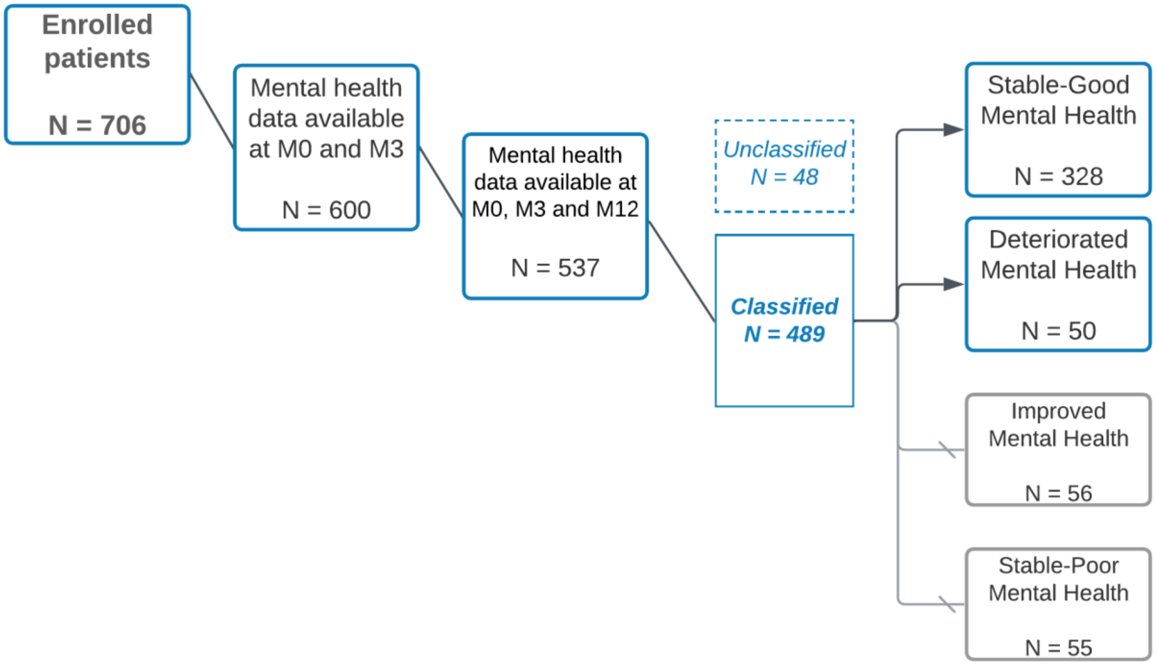


Figure S1. Study flowchart.

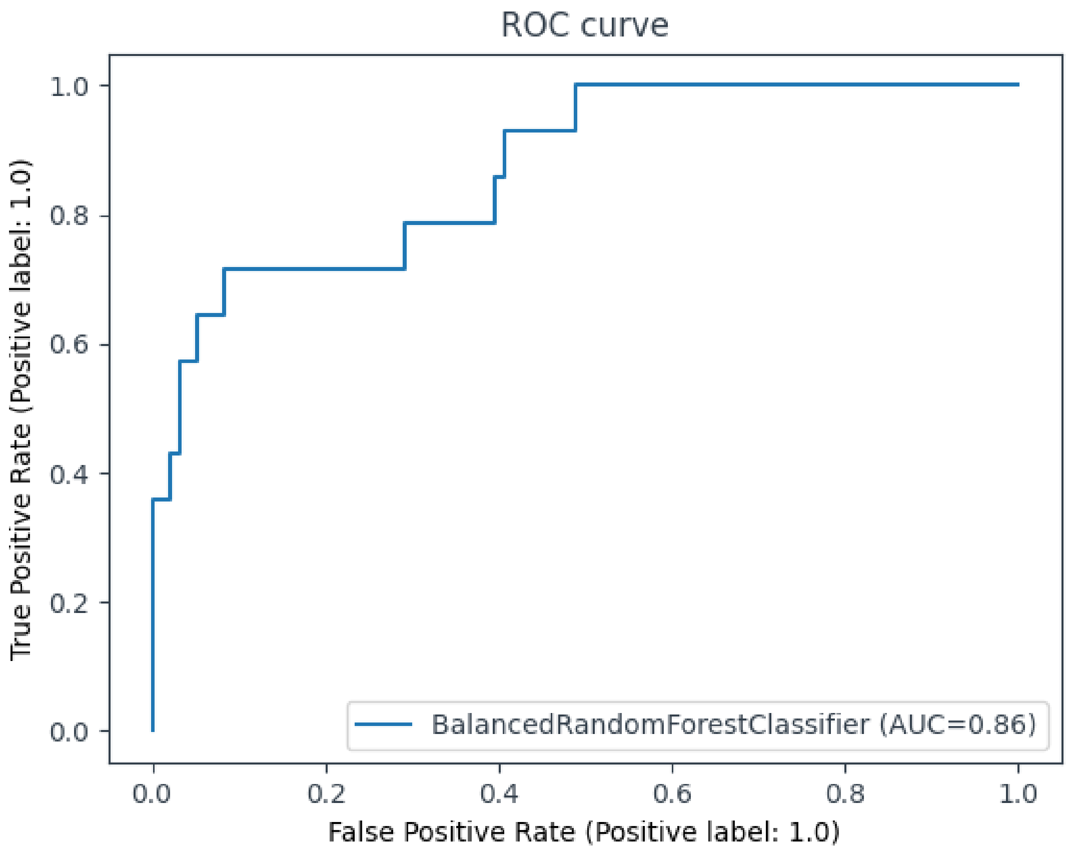


Figure S2. Receiver Operating Characteristic Curve for discriminating between Deteriorating and Stable-Good Mental health groups according to the cross-validated Model 1.

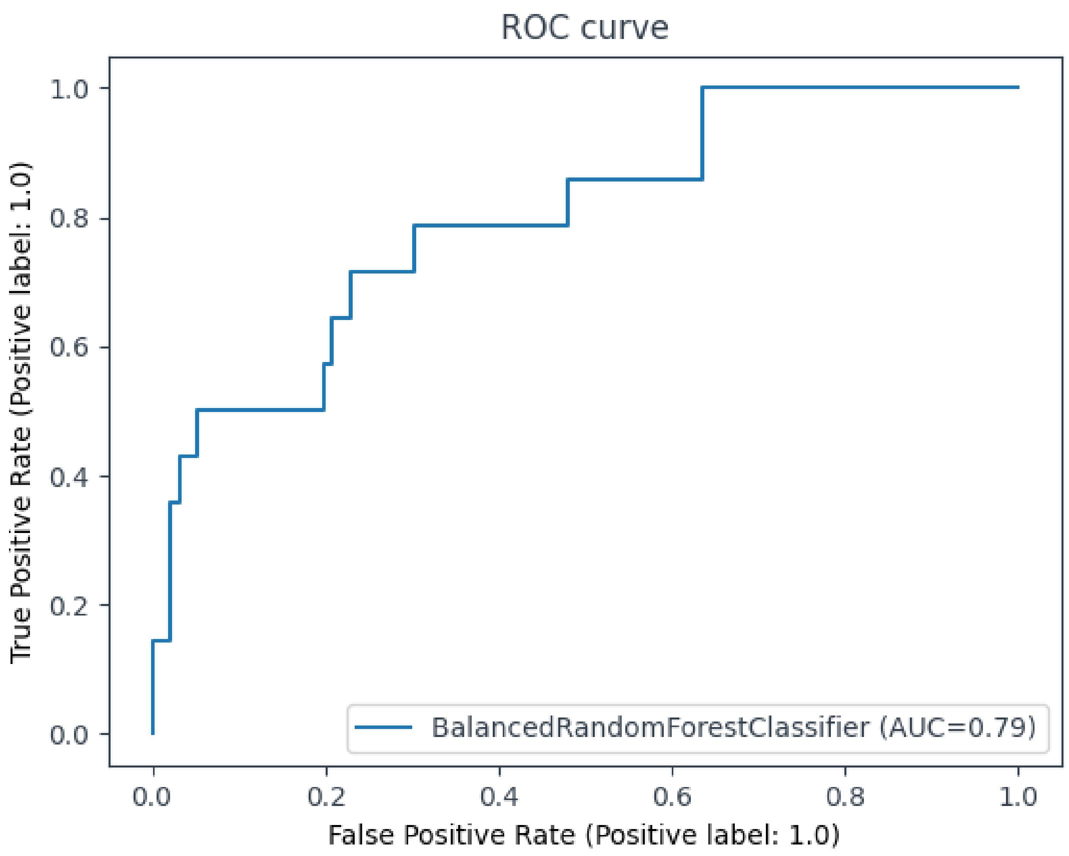


Figure S3. Receiver Operating Characteristic Curve for discriminating between Deteriorating and Stable-Good Mental health groups according to the cross-validated Model 2.

Table S1. Model performance as a function of the imputation method used to address missing data.

| **Performance metrics** | **Model 1** | | **Model 2** | |
|---|---|---|---|---|
| | Replace with median | Multivariate imputation | Replace with median | Multivariate imputation |
| **Specificity** | 0.866 ± 0.01 | 0.863 ± 0.01 | 0.759 ± 0.0 | 0.756 ± 0.01 |
| **Sensitivity** | 0.846 ± 0.01 | 0.845 ± 0.0 | 0.747 ± 0.0 | 0.741 ± 0.02 |
| **Accuracy** | 0.790 ± 0.0 | 0.788 ± 0.0 | 0.747 ± 0.03 | 0.741 ± 0.02 |
| **Precision** | 0.845 ± 0.0 | 0.844 ± 0.0 | 0.750 ± 0.0 | 0.742 ± 0.02 |
| **F1** | 0.542 ± 0.0 | 0.539 ± 0.01 | 0.401 ± 0.01 | 0.388 ± 0.04 |
| **AUC** | 0.864 ± 0.0 | 0.878 ± 0.01 | 0.790 ± 0.0 | 0.799 ± 0.0 |

## References


1 Wu, Y. *et al.* Accuracy of the Hospital Anxiety and Depression Scale Depression subscale (HADS-D) to screen for major depression: systematic review and individual participant data meta-analysis. *bmj* **373** (2021).

2 Vodermaier, A. & Millman, R. D. Accuracy of the Hospital Anxiety and Depression Scale as a screening tool in cancer patients: a systematic review and meta-analysis. *Supportive Care in Cancer* **19**, 1899-1908 (2011).

3 Watson, D., Clark, L. A. & Tellegen, A. Development and validation of brief measures of positive and negative affect: the PANAS scales. *Journal of personality and social psychology* **54**, 1063 (1988).

4 Simard, S. & Savard, J. Fear of Cancer Recurrence Inventory: development and initial validation of a multidimensional measure of fear of cancer recurrence. *Supportive care in cancer* **17**, 241-251 (2009).

5 Aaronson, N. K. *et al.* The European Organization for Research and Treatment of Cancer QLQ-C30: a quality-of-life instrument for use in international clinical trials in oncology. *JNCI: Journal of the National Cancer Institute* **85**, 365-376 (1993).

6 Heitzmann, C. A. *et al.* Assessing self-efficacy for coping with cancer: Development and psychometric analysis of the brief version of the Cancer Behavior Inventory (CBI-B). *Psycho-Oncology* **20**, 302-312 (2011).

7 Watson, M. *et al.* The Mini-MAC: further development of the mental adjustment to cancer scale. *Journal of Psychosocial Oncology* **12**, 33-46 (1994).

8 Bonanno, G. A., Pat-Horenczyk, R. & Noll, J. Coping flexibility and trauma: the perceived ability to cope with trauma (PACT) scale. *Psychological Trauma: Theory, Research, Practice, and Policy* **3**, 117 (2011).

9 Morrill, E. F. *et al.* The interaction of post-traumatic growth and post-traumatic stress symptoms in predicting depressive symptoms and quality of life. *Psycho-Oncology: Journal of the Psychological, Social and Behavioral Dimensions of Cancer* **17**, 948-953 (2008).

10 Moser, A., Stuck, A. E., Silliman, R. A., Ganz, P. A. & Clough-Gorr, K. M. The eight-item modified Medical Outcomes Study Social Support Survey: psychometric evaluation showed excellent performance. *Journal of clinical epidemiology* **65**, 1107-1116 (2012).

11 Rocchi, S. *et al.* The Walsh family resilience questionnaire: the Italian version. *Neuropsychiatric disease and treatment* **13**, 2987 (2017).

12 Campbell-Sills, L. & Stein, M. B. Psychometric analysis and refinement of the connor–davidson resilience scale (CD-RISC): Validation of a 10-item measure of resilience. *Journal of Traumatic Stress* **20**, 1019-1028 (2007).

13 Garnefski, N. & Kraaij, V. Cognitive emotion regulation questionnaire–development of a short 18-item version (CERQ-short). *Personality and individual differences* **41**, 1045-1053 (2006).

14 Brown, K. W. & Ryan, R. M. The benefits of being present: mindfulness and its role in psychological well-being. *Journal of personality and social psychology* **84**, 822 (2003).

15 Scheier, M. F., Carver, C. S. & Bridges, M. W. Distinguishing optimism from neuroticism (and trait anxiety, self-mastery, and self-esteem): a reevaluation of the Life Orientation Test. *Journal of personality and social psychology* **67**, 1063 (1994)

16 van Buuren, S., Groothuis-Oudshoorn, K. mice: Multivariate Imputation by Chained Equations in R. Journal of Statistical Software **45**, 1-67 (2011).

17 Little, R.J.A., Rubin, D.B. Statistical Analysis with Missing Data. John Wiley & Sons, Inc., New York, NY, USA (1986).
